# The smoothened agonist SAG reduces mitochondrial dysfunction and neurotoxicity of frataxin-deficient astrocytes

**DOI:** 10.1186/s12974-022-02442-w

**Published:** 2022-04-12

**Authors:** Andrés Vicente-Acosta, Alfredo Giménez-Cassina, Javier Díaz-Nido, Frida Loria

**Affiliations:** 1 Centro de Biología Molecular Severo Ochoa (CSIC-UAM), Nicolás Cabrera 1, 28049 Madrid, Spain; 2 Departamento de Biología Molecular, Universidad Autónoma de Madrid, Francisco Tomás y Valiente, 7, Ciudad Universitaria de Cantoblanco, 28049 Madrid, Spain; 3 Instituto de Investigación Sanitaria Puerta de Hierro, Segovia de Arana, Hospital Universitario Puerta de Hierro, Joaquín Rodrigo 1, Majadahonda, 28222 Madrid, Spain; 4 Program in Molecular Biosciences, Doctoral School, Universidad Autónoma de Madrid, Madrid, Spain; 5 Laboratorio de Apoyo a la Investigación, Hospital Universitario Fundación Alcorcón, Budapest 1, Alcorcón, 28922 Madrid, Spain

**Keywords:** Frataxin, Reactive astrocytes, Sonic hedgehog, Smoothened agonist, Mitochondrial dysfunction, Neurotoxicity

## Abstract

**Background:**

Friedreich’s ataxia is a rare hereditary neurodegenerative disease caused by decreased levels of the mitochondrial protein frataxin. Similar to other neurodegenerative pathologies, previous studies suggested that astrocytes might contribute to the progression of the disease. To fully understand the mechanisms underlying neurodegeneration in Friedreich’s ataxia, we investigated the reactivity status and functioning of cultured human astrocytes after frataxin depletion using an RNA interference-based approach and tested the effect of pharmacologically modulating the SHH pathway as a novel neuroprotective strategy.

**Results:**

We observed loss of cell viability, mitochondrial alterations, increased autophagy and lipid accumulation in cultured astrocytes upon frataxin depletion. Besides, frataxin-deficient cells show higher expression of several A1-reactivity markers and release of pro-inflammatory cytokines. Interestingly, most of these defects were prevented by chronically treating the cells with the smoothened agonist SAG. Furthermore, in vitro culture of neurons with conditioned medium from frataxin-deficient astrocytes results in a reduction of neuronal survival, neurite length and synapse formation. However, when frataxin-deficient astrocytes were chronically treated with SAG, we did not observe these alterations in neurons.

**Conclusions:**

Our results demonstrate that the pharmacological activation of the SHH pathway could be used as a target to modulate astrocyte reactivity and neuron–glia interactions to prevent neurodegeneration in Friedreich’s ataxia.

**Supplementary Information:**

The online version contains supplementary material available at 10.1186/s12974-022-02442-w.

## Background

Friedreich´s ataxia (FRDA) is a neurodegenerative disease, with an autosomal recessive inheritance pattern, for which there is still no cure. It is characterized by a developmental hypoplasia affecting the spinal cord and medulla, and a neurodegenerative process mainly affecting the cerebellum. The progressive cerebellar damage leads to loss of movement coordination and equilibrium in FRDA patients [1, 2]. In up to 95% of cases, FRDA is caused by an expansion of a guanine–adenine–adenine (GAA) triplet located in the first intron of the *FXN* gene, causing a significant reduction in the transcription levels of the mitochondrial protein frataxin (FXN) [3].

Classically, the study of the pathophysiology of neurodegenerative diseases has been focused on untangling the processes and molecular mechanisms where neurons are involved. However, significant evidence has demonstrated the important role that glial cells have as active contributors to the neurodegenerative process associated with different neurodegenerative diseases [4–8]. Among all glial cells, astrocytes constitute a high percentage of cells in the mammalian central nervous system (CNS), providing trophic support to neurons, promoting synapse formation and functioning, protecting neurons against oxidative stress and participating in the propagation of action potentials [9, 10]. These functions are executed mainly by secreted proteins, which means that astrocytes and neurons are in constant communication. Even so, as these cells are essential for CNS homeostasis, they are also key players in brain dysfunction [11–13].

In response to different stimuli, astrocytes undergo a process called reactive astrocytosis, having important changes in gene expression and functionality [14]. Neuroinflammation and ischemia induce the formation of at least two types of reactive astrocytes, the pro-inflammatory and neurotoxic astrocytic phenotype A1, and the neuroprotective astrocytic phenotype A2. A1-reactive astrocyte formation is triggered by the release of a cytokine cocktail from activated microglia, comprising interleukin 1 alpha (IL-1α), tumor necrosis factor alpha (TNF-α) and the complement component C1q [14]. Blocking the release of these cytokines has been observed to be neuroprotective in different models of Parkinson´s disease and retinal injury [15, 16]. Importantly, A1-reactive astrocytes are present in post-mortem tissues from patients with different neuroinflammatory and neurodegenerative diseases and in in vitro models of neuroinflammatory conditions, which suggests that they might have a crucial role in the neuropathology of these diseases [14, 15, 17–20].

In FRDA, some works using in vivo models of *FXN* silencing in glial cells have shown that this protein is essential for cell survival and neuronal functioning. In *Drosophila melanogaster,* the specific knockdown of *FXN* in glial cells generates a similar phenotype to the one observed after silencing *FXN* ubiquitously: increased neuronal degeneration, altered locomotor activity and reduced lifespan [21]. Moreover, we previously reported that human astrocytes (HAs) with reduced FXN levels have decreased viability and proliferation, and released soluble factors that altered astrocyte–neuron cross talk and cause neuronal toxicity [22]. Still, an in-depth characterization of the reactivity phenotype of astrocytes, if A1 or A2, has not been made yet in FRDA models, nor has it been studied whether pharmacological agents could be used to prevent glial reactivity and/or its associated neurotoxicity in this model.

As mentioned, there is no cure or approved treatment for FRDA. However, numerous therapeutic approaches targeting different stages of the disease are being developed [23]. Recent reports have shown that the activation of the Sonic hedgehog (SHH) signaling pathway using SHH agonists like the smoothened agonist (SAG), confers astrocyte-mediated neuroprotection from kainate-induced cell death [24]. SHH belongs to a family of signaling molecules with critical roles in development and cell cycle regulation [25]. Under homeostatic conditions, SHH is released principally by neurons, being a key factor in neuron–astrocyte signaling by helping keep astrocytes in a non-reactive state [25–27]. However, after CNS injury, astrocytes can release SHH. As they are responsive to this molecule, it is possible that SHH acts in a paracrine manner at least in astrocytes, having detrimental or beneficial effects for neurons and themselves depending on the insult [28, 29]. Thus, apart from being crucial for brain formation, the SHH cascade may have a fundamental role in the adult brain, mainly by regulating neuron–glia interactions [27], which makes it an interesting target to reduce/limit astrocyte-mediated neurotoxicity in FRDA.

In the present work, we show the effects of *FXN* silencing on HA features and functionality. Our data demonstrate that in vitro* FXN* knockdown in HAs reduced cellular survival, altered mitochondrial activity and dynamics, and polarized these cells towards a harmful reactivity profile predominantly consistent with an A1-like phenotype. We also found that the chronic treatment with SAG restored several of the morphological and functional alterations induced by the lack of FXN. More importantly, SAG positively modulated neuron–glia interactions by reverting the neurotoxicity induced by FXN-deficient astrocytes on mouse cortical neurons, increasing neuronal viability, neurite length and synapse formation. In summary, our results show the importance of FXN for normal astrocyte function and the therapeutic potential of pharmacologically targeting the SHH signaling pathway on glial cells to attenuate neurodegenerative processes associated with FRDA disease progression.

## Methods

### Lentiviral production and titration

The in vitro model of *FXN* knockdown in HAs was generated using a short hairpin RNA (shRNA) packaged in a lentiviral vector (LV). For LV production, we used three plasmids: the packaging plasmid pCMVdR8.74 (Addgene, Cat. No. 12259), the envelope plasmid pMD2.G (Addgene, Cat. No. 22036) and the plasmid containing the gene of interest. To specifically knockdown FXN, we used a plasmid containing the sequence against the human *FXN* gene (shRNA37, Sigma Mission® shRNA, Cat. No TRCN0000006137) and a plasmid containing a non-specific scrambled sequence (Addgene, Cat. No. 1864) was used as control. LV packaging and titration was carried out following standard protocols already tested in previous works of this laboratory [30, 31]. Briefly, viral packaging was carried out by transfecting the human embryonic kidney 293T (HEK293T) cell line with the three plasmids using Lipotransfectin (Solmeglas, Cat. No. SBM0959), following the manufacturer’s instructions. After 48 h, the supernatant was collected and filtered with a low-binding protein 0.45-μm filter (Millipore, Cat. No. SLHVM33RS), aliquoted and stored at − 80 °C. Virus titer was estimated by flow cytometry in HEK293T cells transduced with LV expressing the GFP protein. For this, at 72 h post-transduction, cells were fixed with 1% paraformaldehyde (PFA) in phosphate-buffer saline (PBS) containing 2% fetal bovine serum (FBS, Sigma, Cat. No. F7524) and analyzed at the FACSCalibur (BD Biosciencies). As all LVs were produced in parallel, we assumed that all of them had the same titer as the one expressing GFP.

### Cell cultures

*Cell line cultures﻿:* HEK293T cells were cultured in Dulbecco’s modified Eagle medium (DMEM) supplemented with 10% heat-inactivated FBS, 2 mM l-glutamine and (100 IU/mL, 100 µg/mL) penicillin/streptomycin. Cell cultures were maintained at 37 °C in a humidified incubator with 5% of CO_2_ and 95% humidity.

*Human astrocyte cultures:* Healthy cortical fetal human astrocytes (HAs) were obtained from ScienCell Research Laboratories (ScienCell, Cat. No. 1800) and cultured in complete astrocyte medium (ScienCell, Cat. No. 1801). Between 5000 and 7500 cells/cm^2^ cells were plated in 15 μg/ml poly-l-lysine-coated (Sigma, Cat. No. P2636) culture dishes (100 mm). When confluent, cells were detached using 0.25% trypsin/0.5 mM ethylenediaminetetraacetic acid (EDTA) and seeded in 100-mm dishes or in multi-well plates (with or without coverslips) pre-treated with poly-l-lysine at a density between of 5000 and 7500 cells/cm^2^ cells.

*Mouse primary cortical neuron cultures:* Primary cortical neurons were obtained from C57BL/6 mice at embryonic day 17–18, as previously described [32]. Pregnant mice were killed with CO_2_ and the fetuses were removed and decapitated. The dissection was carried out in complete Hank´s Balanced Salt Solution (HBSS, ThermoFisher, Cat. No. 24020117). Then, cortices were washed with HBSS without Ca^2+^ and Mg^2+^ (ThermoFisher, Cat. No. 14170112), digested in Trypsin (0.25%: Sigma, Cat. No. T4549) containing DNase I (1 mg/ml: Roche Diagnosis, Cat. No. 10104159001) for 15 min at 37 °C, and mechanically dissociated with a fire-polished Pasteur pipette. Cortical cells were seeded in 200 μg/ml poly-l-lysine-coated plates at a density of 40,000–60,000 cells/cm^2^, and maintained in neuronal medium (Neurobasal medium (Life Technologies, Cat. No. 21103-049), 2% B27 (Life Technologies, Cat. No. 17504-044), 1% GlutaMAX (Life Technologies, Cat. No. 35050-038) and (100 IU/mL, 100 µg/mL) penicillin/streptomycin). Cells were then incubated at 37 °C in a humidified atmosphere containing 5% of CO_2_.

### Drug treatments

Cells were transduced with the LV at a multiplicity of infection (MOI) of 10, keeping them in culture for 48, 72 or 96 h. For the treatment with SAG, astrocytes were treated daily with SAG (EMD Millipore Corp., Cat. No. 566660) at 1 μM for 6 consecutive days. As a positive control of A1-reactivity, astrocytes were treated for 24 h with TNF-α (Cell Signaling Technology, Cat. No. 8902), IL-1α (Sigma, Cat. No. SRP6295) and C1q (MyBioSource, Cat. No. MBS143105) at a concentration of 30 ng/ml, 3 ng/ml and 400 ng/ml, respectively. TNF-α and IL-1α were diluted in PBS and stored at − 20 °C and C1q was diluted in H_2_O and stored at 4 °C. For the autophagy experiments, astrocytes were treated with 50 nM bafilomycin A1 (BafA1, ChemCruz, Cat. No. sc-201550A) for 4 h at 37 °C.

### Preparation of astrocyte conditioned medium

Astrocyte conditioned medium (ACM) was obtained by plating HAs in 6-well culture dishes at a density between 5000 and 7500 cells/cm^2^ in complete astrocyte medium. 48 h after plating, HAs were transduced either with the LV-scrambled or the LV-shRNA37, replacing the astrocyte medium with neuronal medium 48 h after transduction, and leaving the cells in neuronal medium for further 48 h, a total of 96 h post-transduction. To chronically treat the shRNA37-transduced HAs with SAG, we added the compound daily for 6 consecutive days starting immediately after plating. At the end of the experiments, ACM from each experimental condition was collected and centrifuged three times (200 *g* × 5 min, 1000 *g* × 10 min and 20,000 *g* × 25 min), then filtered with a low-binding protein 0.45-μm filter and stored at − 20 °C. To treat neurons with these conditioned media, cells were plated in neuronal medium for 3 h and then shifted to ACM and maintained under these conditions for 5 days.

### Cell viability assays

*Cell metabolic activity assay (MTS):* Cellular metabolic activity was measured using the kit CellTiter 96® AQueous One Solution Cell Proliferation Assay (Promega, Cat. No. G3580). The MTS assay is a colorimetric method based on the conversion of a tetrazolium compound into a colored formazan product by dehydrogenase enzymes in metabolically active cells that can be quantified by measuring the absorbance at 490 nm. For these experiments, cells were plated in 96-well culture dishes, adding MTS at a final concentration of 317 μg/ml at the end of the treatments. After 1–2 h of incubation at 37 °C, the absorbance was measured using the Dynex Opsys MR microplate reader (Dynex technologies).

*Calcein acetoxymethyl ester/propidium iodide (calcein AM/PI) uptake:* Cell viability was measured according to previously described methods [33]. Briefly, the non-fluorescent calcein is transformed into a green-fluorescent compound in living cells due to the hydrolysis by intracellular esterases. In non-viable cells, the damaged plasma membrane allows the PI to reach the nucleus and intercalate into the double-stranded DNA, emitting red fluorescence. Thus, cells were incubated with a mixture of 1 μM calcein AM (ThermoFisher, Cat. No. C3100MP) and 2 μM of PI (Sigma, Cat. No. P4170) for 30 min at 37 °C. Images from at least 15 randomly selected fields per experimental condition and biological replicate were acquired with an Axiovert200 (Zeiss) fluorescence inverted microscope coupled to a monochrome sCMOS camera (Hamamatsu). The percentage of non-viable cells (PI + cells) was calculated as the percentage of red cells relative to the total number of cells (both red and green).

### Flow cytometry

*Mitochondrial superoxide production:* The production of superoxide in the mitochondria was measured by flow cytometry using the MitoSOX™ Red probe (ThermoFisher, Cat. No. M36008). This compound penetrates into the mitochondria of living cells, where it is rapidly oxidized by mitochondrial superoxide and transformed into a fluorescent product. For that, culture medium was removed from the plate and replaced with medium containing 5 μM MitoSOX™ Red probe for 30 min at 37 °C. Then, cells were collected by trypsinization and centrifuged at 800 *g* for 5 min. The pellets were washed twice with HBSS and resuspended in 500 μl of FACS binding buffer, consisting in HBSS containing Ca^2+^ and Mg^2+^, and 1% bovine serum albumin (BSA: Roche Diagnostics, Cat. No. 10735094001). Cell suspensions were transferred to cytometry tubes and analyzed in a FACSCanto II flow cytometer (BD Biosciences) with 488 nm excitation, measuring MitoSOX™ Red probe in the FL2/3 and analyzing 10,000 events per condition. The mean intensity (FL2) of each population was analyzed and plotted using the FlowJo 10.7 software.

*Lipid droplet accumulation:* The presence of lipid droplets in the cytoplasm of HAs was measured by flow cytometry using the BODIPY 493/503 dye (ThermoFisher, Cat. No. D3922). For this, culture medium was removed and replaced with astrocyte medium without FBS containing 800 nM BODIPY 493/503 for 1 h at 37 °C. Then, cells were collected by trypsinization and centrifuged at 800 *g* for 5 min. The pellets were washed twice with HBSS and resuspended in 500 μl of FACS binding buffer. Cell suspensions were transferred to cytometry tubes and analyzed in a FACSCanto II flow cytometer with 488 nm excitation, measuring BODIPY 493/503 probe in the FL1 and analyzing 10,000 events per condition. The mean intensity (FL1) of each population was analyzed and plotted using the FlowJo 10.7 software.

### Mitochondrial respiration analysis

Mitochondrial respiration was analyzed using the Seahorse XF24 Extracellular Flux Analyzer (Agilent), which measures the oxygen consumption rate (OCR) in intact cultured cells [34, 35]. HAs from all experimental conditions were collected, and 30,000 cells per well were re-plated in the seahorse plate 24 h before the assay. The experiment was carried out in DMEM supplemented with 5 mM glucose and 1 mM sodium pyruvate, without sodium bicarbonate. After determination of a respiratory baseline, we sequentially applied the following drugs: The mitochondrial ATP synthase inhibitor oligomycin (0.5 μM; Millipore. Cat. No. 495455), to determine the fraction of ATP-coupled mitochondrial respiration; the ionophore uncoupler carbonyl cyanide 4-(trifluoromethoxy) phenylhydrazone (FCCP; 0.5 μM: Sigma, Cat. No. C2920), to determine maximal respiratory capacity by dissipating the mitochondrial proton gradient and imposing a situation of high energy demand; and finally, the mitochondrial complex III inhibitor antimycin A (4 μM; Sigma, Cat. No. A8674) was used to block mitochondrial respiration. OCR values obtained with the pharmacological manipulation described above were used to calculate different mitochondrial respiration parameters: basal respiratory capacity, maximal respiratory capacity, spare respiratory capacity, proton leak and ATP-coupled respiration.

### Mitochondrial network analysis

Mitochondria were stained with Mitotracker Red (ThermoFisher, Cat. No. 7513) and analyzed with the ImageJ plugin “Mitochondrial network analysis” (MiNA) [36]. Before fixing, 2 μM Mitotracker Red was added to the cells for 30 min at 37 °C. Then, randomly selected fields were captured using a LSM510 vertical laser scanning confocal microscope (Zeiss) and the mitochondrial network from at least 30 cells per experimental condition and biological replicate from each experiment were analyzed with the plugin MiNA.

### Sample collection and RNA extraction

After treatments, cells were washed twice with PBS, frozen on dry ice and stored at − 80 °C until processed. Total RNA was extracted using the NZY Total RNA Isolation kit (NZYTech, Cat. No. MB13402) following the manufacturer’s instructions. The concentration and purity of the RNA was analyzed using a NanoDrop One/One^C^ spectrophotometer (ThermoFisher).

### Reverse transcription and quantitative PCR (RT-qPCR)

First strand cDNA was synthesized from 0.5 μg of total RNA (10 ng/μl final concentration) using the NZY First-Strand cDNA Synthesis kit (NZYTech, Cat. No. MB12501) following the manufacturer’s instructions. Primers were designed from previously published sequences or in our house (Additional file 1: Table S1). To avoid genomic DNA amplification, primers have an intron-spanning design (all from ThermoFisher). qPCR was performed using 12 ng of cDNA using the Luminaris Color HiGreen High ROX qPCR Master Mix (ThermoFisher, Cat. No. K0362) and 500 nM primers using the 7900HT Fast Real-Time PCR System (ThermoFisher) with the following cycling conditions: 50 °C for 2’ + 95 °C for 10’; 40 cycles of 95 °C for 15” and 60 °C for 1’; 95 °C for 15” + 60 °C for 15” + 95 °C for 15”. The cycle threshold (Ct) value for each gene was normalized to the Ct value of the housekeeping gene glyceraldehyde-3-phosphate dehydrogenase (*GAPDH*). Relative expression values were calculated with the comparative Ct method [37]. Results are expressed as fold change of the LV-scrambled condition.

### Protein extraction

Cells were washed once with PBS and stored at − 80 °C until protein extraction. Once thawed, cells were homogenized in ice-cold RIPA lysis buffer: 50 mM Tris–HCl pH 7.6, 150 mM sodium chloride (NaCl), 1% Triton X-100, 0.5% sodium deoxycholate and 0.1% sodium dodecyl sulfate (SDS), protease inhibitors (Roche Diagnostics, Cat. No. 11697498001) and 1 μM okadaic acid (phosphatases inhibitor; Sigma, Cat. No. 459618). Then, samples were sonicated and centrifuged at 16,000 *g* for 10 min at 4 °C. Protein concentration was measured with the BCA protein assay kit (ThermoFisher, Cat. No. 23227), posteriorly denaturing the samples with SDS 5 × loading buffer for 5 min at 100 °C.

### Western blot

For immunoblotting, equal amounts of protein (10 μg) were separated using pre-cast gradient 4–12% acrylamide–bisacrylamide gels (Invitrogen, Cat. No. NP0322BOX) and transferred to nitrocellulose membranes (Invitrogen, Cat. No. IB23002) using the dry blotting system iBlot 2 (Invitrogen, Cat. No. IB21001), following the manufacturer’s instructions. Then, membranes were blocked in a solution of non-fat dried milk at 5% in PBS 0.1% Tween-20 (Sigma, Cat. No. 822184) or BSA at 2% in PBS 0.1% Tween-20 for 30 min. Blocked membranes were incubated overnight at 4 °C with primary antibodies. Membranes were then washed with PBS 0.1% Tween-20 and incubated with the corresponding horseradish peroxidase-conjugated secondary antibody (1:5000, Southern Biotech Assoc. Inc) for 1 h at room temperature. Antibody binding to the proteins of interest was visualized by chemiluminescence using the Amersham ECL prime detection reagent (GE Healthcare Life Sciences, Cat. No. RPN2232). The quantification of the intensity of the protein bands was performed using the ImageJ software, and protein values were normalized to those obtained from the housekeeping protein β-Actin, used as a loading control. Primary antibodies used in this study were: mouse anti-β-actin (1:5000; Sigma, Cat. No. A5441), mouse anti-frataxin (1:1000; Abcam, Cat. No. ab110328), rabbit anti-C3 (1:1000; Abcam, Cat. No. ab97462), rabbit anti-cleaved PARP1 (1:1000; Cell Signaling, Cat. No. 9541), mouse anti-p53 (1:1000; BD Biosciences, Cat. No. 554294), rabbit anti-p21 (1:1000; Santa Cruz, Cat. No. sc397), rabbit anti-phospho-Drp1 (Ser616) (1:1000; Cell Signaling, Cat. No. 3455), mouse anti-Drp1 (1:1000; BD Biosciences, Cat. No. 611113), total OXPHOS rodent WB antibody cocktail (1:500; Abcam, Cat. No. ab23707), rabbit anti-LC3 (1:1000; Sigma, Cat. No. L7543), rabbit anti-PINK1 (1:1000; Abcam, Cat. No. ab23707), mouse anti-Parkin (1:1000; Abcam, Cat. No. ab77924), mouse anti-OPA1 (1:1000; BD Biosciences, Cat. No. 612606), mouse anti-MFN1 (1:1000, Abcam, Cat. No. ab126575), mouse anti-MFN2 (1:1000; Abnova, Cat. No. H00009927-M01), rabbit anti-PGC-1α (1:1000; Santa Cruz, Cat. No. sc13067) and rabbit anti-perilipin-2 (1:1000; Invitrogen, Cat. No. PA5-29099).

### Wound healing assay

Astrocytes were grown to a confluent monolayer in 6-well plates, while submitted to the corresponding treatment. Once cells were confluent, a 1000-μl pipette tip was used to scratch through the surface from top to bottom of the well. The plate was previously marked to identify and capture the same region of the scratch. Then, cells were incubated for 30 min with 1 μM Calcein AM, and observed with an Axiovert200 (Zeiss) fluorescence inverted microscope coupled to a monochrome sCMOS camera (Hamamatsu), acquiring images right after the scratch (0 h) and 24 h later. For each experimental condition, 8 images of at least 4 independent wounds per biological replicate were taken. To measure wound width, the distance between wound edges was measured several times along the wound to estimate the mean width of each wound using the ImageJ software. For each experimental condition, wound closure was calculated as wound with at 0 h minus wound with at 24 h.

### Immunocytochemistry, immunostaining and image analysis

Cells were plated in multi-well dishes containing glass coverslips and sequentially fixed with 2% PFA for 15 min followed by 4% PFA for 15 min. After washing with cold PBS, cells were incubated for 20 min in blocking solution containing 1% BSA and 0.1% Triton X-100 in PBS. Then, cells were incubated with the primary antibody diluted in the same blocking solution overnight at 4 °C. After that, cells were washed three times with cold PBS and incubated for 1 h at room temperature with the corresponding secondary antibody conjugated to Alexa-488, Alexa-555 or Alexa-647 (1:1000; Molecular Probes) diluted in blocking solution. The nuclei were stained with To-Pro3 (1:5000; ThermoFisher, Cat. No. T3605), mounting the coverslips on slides using Fluoromount G (Southern Biotech Assoc. Inc, Cat. No. 0100-01). Non-specific interactions of secondary antibodies were verified by omitting the primary antibodies. Finally, images from at least 10 different randomly selected fields per experimental condition and biological replicate were acquired using a LSM510 vertical laser scanning confocal microscope (Zeiss) or an Axiovert200 (Zeiss) fluorescence inverted microscope coupled to a monochrome sCMOS (Hamamatsu) camera. Primary antibodies used in this study were: mouse anti-s100β (1:1000; Sigma, Cat. No. AMAB91038), rabbit anti-C3 (1:500; Abcam, Cat. No. ab97462), rabbit anti cleaved caspase 3 (1:400; Cell Signaling, Cat. No. 9661), rabbit anti-phospho-Drp1 (Ser616) (1:300; Cell Signaling, Cat. No. 3455), mouse anti-PSD95 (1:500; EMD Millipore, Cat. No. MABN68), rabbit anti-synaptophysin (1:1000; SynapticSystems, Cat. No. 101002), rabbit anti-MAP2 (1:1000; Abcam, Cat. No. ab32454) and mouse anti-SMI-31 (1:1000; EMD Millipore, Cat. No. NE1022). To quantify the fluorescence intensity of C3 protein staining, we estimated the corrected total cell fluorescence (CTCF) of at least 30 cells per experimental condition and biological replicate from each experiment using ImageJ. The CTCF is calculated using the following formula: integrated density of the cell of interest—(area of the cell of interest x mean fluorescence of the background readings). To label lipid droplets, cells were incubated with 800 nM BODIPY 493/503 for 1 h at 37 °C before performing live-cell imaging.

*Neuronal quantitative analysis:* To analyze neurite outgrowth and branching in primary neuronal cultures, after immunostaining with SMI-31 and MAP2, at least 10 images from each experimental condition and biological replicate were captured from randomly selected fields. Axons (SMI-31) [38] and dendrites (MAP2) [39] were traced from the cell body to the end of their prolongations, calculating the sum of the distance per field with the ImageJ plugin NeuronJ and normalizing to the number of cells/field [40]. In the case of total neuron number, we counted all DAPI positive cells per field and condition.

*Synapse quantification:* To measure synapse formation in primary neuronal cultures, after immunostaining with the presynaptic marker synaptophysin and the postsynaptic marker PSD-95, synapses from at least 15 different neurons were counted for each experimental condition and biological replicate. For this, 6 randomly selected neurites of the same size (15 μm) were analyzed per cell. Those randomly selected neurites followed only one criterion, they had to be located at a distance of at least one cell body from the soma. The co-localization between synaptophysin and PSD-95 puncta was measured using the JACoP plugin in ImageJ [41].

### Ethical considerations

All studies involving animals were performed with approval from the regional animal ethics committee of Comunidad de Madrid, Spain (protocol numbers PROEX 184/15 and PROEX 013.0/21), according to institutional and European guidelines for animal handling and research (EU Directive 2010/63/EU for animal experiments). All efforts were made to minimize the number and suffering of used animals.

### Statistical analyses

Data from at least three independent experiments are presented as mean ± standard error of the mean (S.E.M) in the figures. Statistical analyses were performed with the GraphPad Prism 7.00 software. In all cases, data were normalized to the corresponding untreated control group and one-sample *t*-tests with a confidence level of 95% were used to compare the untreated and scrambled-transduced groups. We did not find significant differences in any of those comparisons. For the rest of the analyses, for statistical comparisons between groups, we first confirmed that data sets passed the variance homogeneity (Brown–Forsythe) and normality (D’Agostino–Pearson) tests, and then we used one-way ANOVA followed by Tukey’s post hoc test. Statistical significance was attributed when *p* ≤ 0.05.

## Results

### Frataxin expression is downregulated in human astrocytes after shRNA37 transduction

**Fig. 1:**
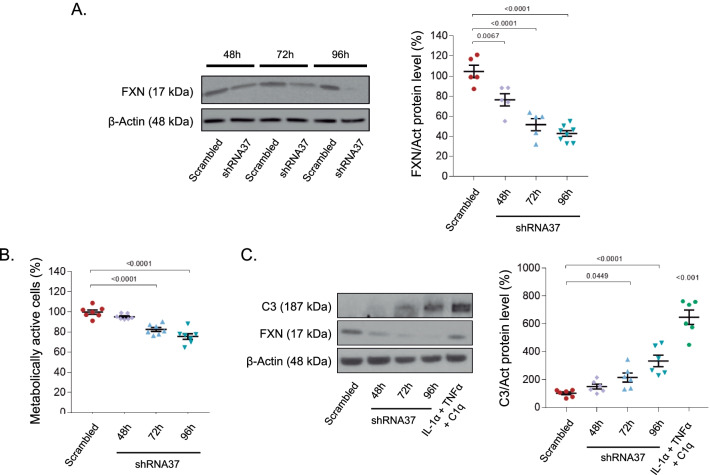
In vitro characterization of *FXN* silencing in HAs*.* **A–C** HAs were transduced with either the LV-scrambled or LV-shRNA37 for the indicated times. **A** Representative immunoblot and quantification of FXN levels over time after transduction. Equal amounts of protein were immunoblotted and probed with antibodies against FXN and β-actin (as loading control) (*n* = 5). **B** Cellular metabolic activity (MTS reduction) in HAs 48, 72 and 96 h post-transduction (*n* = 7). **C** Representative immunoblot and quantification of C3 levels over time after transduction. Equal amounts of protein were immunoblotted and probed with antibodies against C3 and β-actin (as loading control) (*n* = 6). HAs treated for 24 h with IL-1α, TNF-α and C1q were used as a positive control. All values were normalized to the untreated group (data not plotted) of their corresponding time point. Data in all graphs represent mean values ± S.E.M. In **A–C** data were evaluated by one-way ANOVA followed by Tukey’s post hoc test. In **C** data from the IL-1α, TNF-α and C1q treated group were compared to the untreated group (data not plotted) using a one-sample *t*-test

To fully characterize the effects that the lack of FXN has on HAs, their reactivity state and the influence that these cells have on neuronal functioning, we used an in vitro model of *FXN* knockdown. This model is based on the transduction of HAs with a LV encoding for a shRNA, named shRNA37, which reduces both *FXN* mRNA and protein levels. This vector has been validated in our previously published studies [22, 31, 42]. Here, we first characterized the transduction efficiency of the LV-shRNA37, by comparing FXN levels over time between shRNA37-transduced cells and HAs transduced with a LV encoding for a shRNA targeting a scrambled sequence, that was used as control (Fig. 1A). We found a progressive decrease in FXN protein levels that reached similar values as those found in FRDA patients [43, 44] at 96 h post-transduction, being significantly lower than control HAs, as previously observed by our group [22]. As 96 h was the time needed for our iRNA-based model to reach pathological reduction in FXN protein levels, the rest of the experiments of this work were carried out at this time point. Alongside FXN level decline, we detected a progressive reduction in the percentage of metabolically active cells, indicating that FXN deficiency significantly affected astrocyte metabolism (Fig. 1B). In this model, we also studied astrocyte reactivity. In a first approach, as the complement system has been described as a main regulator of astrocyte function [14, 45, 46], we measured protein levels of one of the most upregulated genes in A1 reactive astrocytes, the complement component C3, using HAs treated for 24 h with IL-1α, TNF-α and C1q as positive controls of activation [14] (Fig. 1C). We detected that C3 levels gradually increased in HAs after LV-shRNA37 transduction, albeit less pronounced than in the cells treated with the cytokines, indicating that FXN deficiency induces astrocyte reactivity. Taken together, these results indicate that FXN deficiency reduced cellular metabolism and triggered reactivity in HAs.

### Chronic treatment with SAG diminishes cellular death triggered by *FXN* silencing

**Fig. 2:**
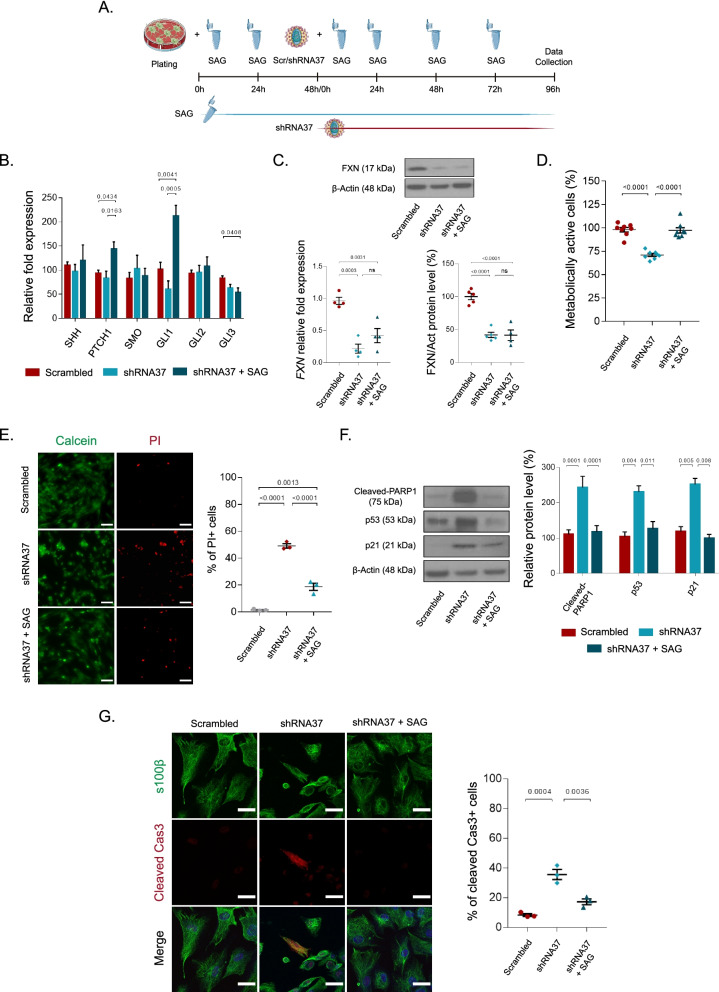
Chronic treatment with SAG improves cell metabolism of FXN-deficient HAs. **A** Schematic representation of SAG chronic treatment of FXN-deficient astrocytes. **B** mRNA expression levels of SHH signaling pathway members were determined by qPCR. Gene expression was normalized to GAPDH and quantified by the comparative Ct method (*n* = 4). **C** The graph on the left shows *FXN* mRNA transcript levels in HAs in all experimental groups, analyzed by qPCR (*n* = 4). The top panel and the graph on the right show a representative immunoblot and quantification of FXN levels in HAs after transduction and treatment with SAG (*n* = 4). **D** Cellular metabolic activity (MTS reduction) in HAs after SAG chronic treatment (*n* = 8). **E** Representative images of live (green) and dead cells (red) estimated by the calcein/PI uptake assay (*n* = 3). HAs transduced and treated with SAG were incubated with both dyes and analyzed by fluorescence microscopy. Scale bars: 200 μm. The graph on the right shows the quantification of cell death, estimated as the % of PI positive cells per field. **F** Representative immunoblots and quantification of cleaved-PARP1, p53 and p21 levels for the indicated groups. Same amounts of protein were immunoblotted and probed with the corresponding antibody and β-actin (as loading control) (*n* = 4). **G** Representative confocal images (left) and quantification (right) of HAs subjected to the indicated experimental conditions (*n* = 3). Cells were fixed and stained with specific antibodies against S100β and cleaved Cas3, and with To-Pro-3 to stain the nuclei (blue). Scale bars: 30 μm. In all graphs, data were normalized to the untreated group (data not plotted) and represent mean values ± S.E.M. In **B–G** data were evaluated by one-way ANOVA followed by Tukey’s post hoc test

Recent studies revealed that activating the SHH pathway in astrocytes with SHH agonists have positive effects in astrocyte function and activity [24]. Thus, we evaluated the effects of the chronic treatment with this molecule in our model of FXN deficiency, a treatment that has been confirmed to have positive effects in experimental models of different pathologies [47–49]. For this, HAs were treated chronically with SAG for 6 days, pretreating the cells for 48 h prior to LV-shRNA37 transduction, and furtherly treating them for 96 h, as indicated in Fig. 2A. To test for the effects of *FXN* silencing and the treatment with SAG in the activation state of the canonical SHH signaling pathway, we evaluated the transcript expression of different downstream effectors and observed that FXN deficiency did not alter any of the evaluated genes of this signaling pathway. However, the chronic treatment of FXN-deficient HAs with SAG significantly increased the expression of the target genes *PTCH1* and *GLI1*, which are target genes of the SHH signaling pathway, demonstrating the activation of this pathway upon chronic treatment with this molecule (Fig. 2B). Besides, chronic treatment with SAG decreased the expression of the repressor gene *GLI3*. We also analyzed *FXN* mRNA and protein levels and found that SAG does not revert the decrease in FXN levels induced by the shRNA37, indicating that an increase in FXN levels by SAG is not the mechanism responsible for any of the effects we could attribute to this molecule in this cell model (Fig. 2C). Since we observed that the lack of FXN in HAs affected cell survival, we measured astrocyte metabolic activity and viability in shRNA37-transduced cells that were co-treated with SAG. In these experimental conditions, we detected that SAG completely restored the metabolic activity of HAs lacking FXN, and significantly diminished cell death (Fig. 2D and E). As FXN deficiency promotes cell death via apoptosis in HAs [22] and in other cell types [42, 50, 51], we examined whether SAG effects on viability were due to a reduction of apoptosis. For this, we evaluated the levels of cleaved PARP1, whose cleavage by caspases is considered a hallmark of apoptosis [52, 53], and p53 and p21 levels, both implicated in DNA repair and cell cycle arrest upon DNA damage, and also in apoptosis [42, 54–56]. In all cases, we observed an increased expression of these proteins when HAs had low FXN levels, but this increase was abolished when the cells were chronically treated with SAG (Fig. 2F). Accordingly, we observed that the number of cleaved caspase 3 (Cas3) positive cells increased in FXN-deficient HAs and decreased after the treatment with SAG, confirming the aforementioned results (Fig. 2G). Overall, these observations indicate that FXN deficiency induces cell cycle arrest and apoptosis in HAs, and that the activation of the SHH pathway with the agonist SAG reverts some of the events that are ultimately affecting cell viability.

### Mitochondrial dysfunction in FXN-deficient astrocytes is rescued after SAG treatment

**Fig. 3:**
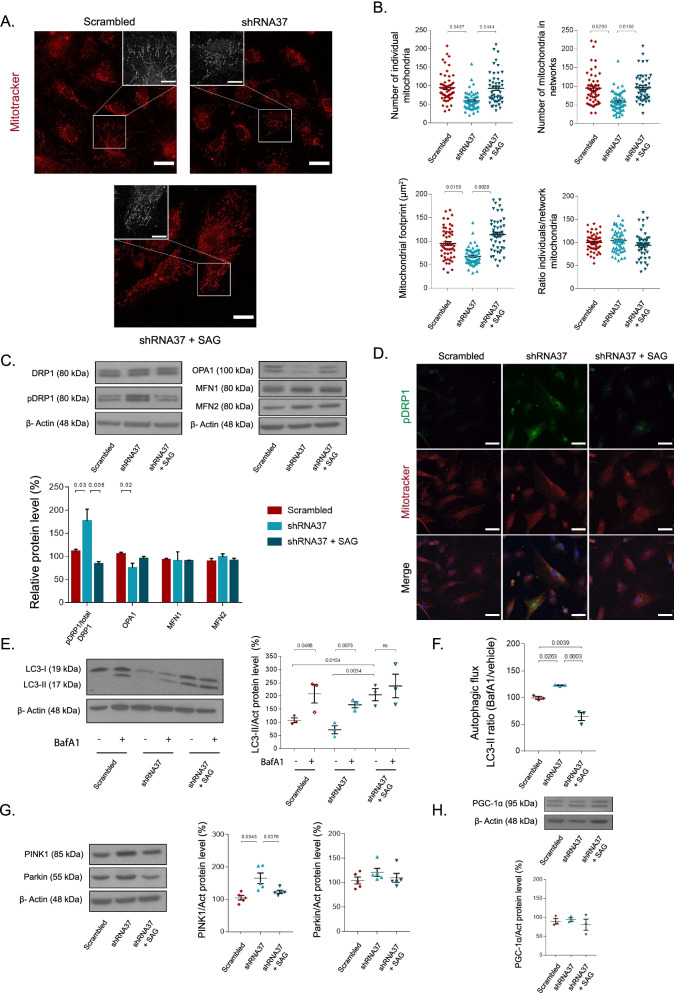
SAG positively modifies mitochondrial morphology and dynamics of FXN-deficient astrocytes. In **A–H**, HAs were transduced with either the LV-scrambled or LV-shRNA37 for 96 h, adding SAG (1 μM) daily for 6 days. **A** Representative confocal images of HAs stained with MitoTracker Red before fixation and imaging. Insets are twofold enlargements of the boxed region. Scale bars: 30 μm (expanded fields) and 15 μm (insets). **B** Analysis of mitochondrial morphology and distribution of MitoTracker Red positive cells using the MiNA plugin (ImageJ). Each plotted value represents a single cell from three independent experiments. **C** Representative immunoblots and quantification of Drp1, pDrp1, OPA1, MFN1 and MFN2 levels across the indicated experimental conditions (*n* = 4). **D** Representative microscopy images of HAs transduced with LV- shRNA37 that were treated or not with SAG. Cells were incubated with MitoTracker Red, fixed and then stained with pDrp1 and with To-Pro-3 to stain the nuclei (blue). Scale bars: 50 μm. **E** Representative immunoblot (left) and quantification (right) of LC3-I and its lipidated form LC3-II in the different experimental conditions before and after treatment with BafA1 (*n* = 3). **F** Quantification of autophagic flux of LC3 calculated as the ratio of LC3-II between BafA1 and vehicle in each experimental condition (*n* = 3). **G** Representative immunoblots and quantifications of PINK1 and Parkin levels in the indicated conditions (*n* = 5). **H** Representative immunoblot and quantification of PGC-1α levels in the different experimental conditions (*n* = 3). Data in all graphs were normalized to the untreated group (data not plotted). Except from **B** where data represent mean ± S.E.M of individual cells across experiments, the rest of the plotted data represent mean values ± S.E.M. In **B**, **C**, **E**–**H** data were evaluated by one-way ANOVA followed by Tukey’s post hoc test

FXN main isoform is located in the mitochondria, where it plays an important role in mitochondrial metabolism, dynamics and redox homeostasis [57–60]. As FXN deficiency leads to important defects in mitochondrial activity and function in different experimental models [61–64], we hypothesized that mitochondrial function and dynamics could be altered in our HAs. Consequently, we examined the potential effects of FXN deficiency on mitochondrial status through different approaches. To evaluate and compare mitochondrial morphology, we used the MitoTracker Red probe in HAs transduced with the LV-shRNA37 that were treated or not with SAG (Fig. 3A). In HAs transduced with the LV-scrambled, the majority of mitochondria were extensively fused, exhibiting the typical filamentous pattern associated with healthy mitochondria. However, mitochondria of FXN-deficient astrocytes exhibited a severely fragmented and swollen morphology. When we analyzed the images with the ImageJ plugin MiNA, we found that both the number of individual mitochondria and mitochondria forming networks were significantly reduced in FXN-deficient HAs, which was confirmed by a decrease in mitochondrial footprint, a measurement of total mitochondrial mass (Fig. 3B). Besides, we did not observe significant changes in the individual mitochondria/networks ratio among the different experimental conditions, indicating a general decrease of mitochondria content in these cells. The analysis also revealed that SAG limited the loss of mitochondrial mass promoted by FXN deficiency (Fig. 3B). In a second approach, we analyzed the expression of different proteins involved in mitochondrial fission (DRP1) and fusion (OPA1 and MFN1/2) [65, 66] across experimental conditions. While MFN1 and MFN2 levels remained unchanged in all cases, we observed a significant increase in the p-DRP1 (active form)/total DRP1 ratio, and a small but significant decrease in OPA1 in shRNA37-transduced HAs, suggesting a shift in mitochondrial dynamics towards an excessive mitochondrial fragmentation in these cells (Fig. 3C). Consistent with the aforementioned changes, we observed an increased number of p-DRP1 positive mitochondria in those cells lacking FXN, which was limited after chronically treating the cells with SAG (Fig. 3D).

As we observed fragmented mitochondria and loss of mitochondrial mass when FXN was almost absent in HAs, we wondered whether these phenomena were possibly due to a decreased mitochondrial biogenesis and/or an increased mitochondrial degradation through autophagy. To identify whether autophagy was activated in HAs in response to FXN deficiency, we evaluated the expression of the autophagy-specific adaptor protein LC3-II and the autophagic flux in FXN-deficient HAs with or without treatment with SAG. To block lysosomal degradation we used BafA1, observing an accumulation of LC3-II in all experimental conditions (Fig. 3E). When we calculated the autophagic flux we found that it was significantly increased in FXN-deficient HAs, and that SAG blocked this process (Fig. 3F). Then, we monitored PINK1 and Parkin protein levels, two key proteins involved in the elimination of mitochondria through mitophagy [67]. As observed in Fig. 3G, while no changes were detected in Parkin levels among all conditions, PINK1 was significantly upregulated in FXN-depleted HAs. Indeed, when these cells were co-treated with SAG, PINK1 levels decreased to similar levels as those observed in control conditions, indicating that *FXN* knockdown induced PINK1/Parkin-mediated mitophagy (Fig. 3G). We also examined the levels of PGC-1α, a protein implicated in mitochondrial biogenesis [68, 69], but we did not observe any changes among experimental conditions (Fig. 3H). These results suggest that *FXN* knockdown in HAs alters mitochondrial dynamics by inducing an excessive number of fission events and reducing mitochondrial mass via enhanced mitophagy, without altering mitochondrial biogenesis, events that are prevented after the chronic treatment with SAG.

**Fig. 4:**
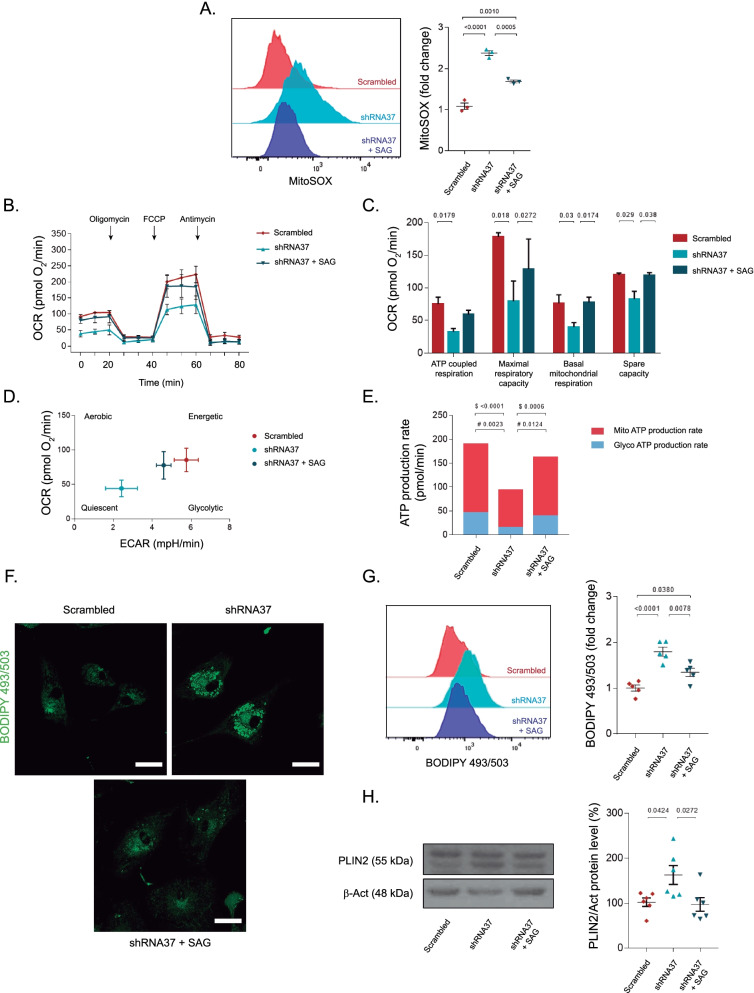
Chronic treatment with SAG improves mitochondrial function and activity of FXN-deficient astrocytes. In **A–H** HAs were transduced with either the LV-scrambled or LV-shRNA37 for 96 h, SAG was added daily at 1 μM for a total of 6 days. **A** Representative histogram and quantification of the flow cytometry analysis of mitochondrial superoxide production using the MitoSOX Red dye (*n* = 3). **B** Analysis of mitochondrial oxygen consumption rates determined in HAs by the Seahorse assay in the indicated experimental conditions. The graph represents the OCR over time before and after the addition of the different drugs that modulate mitochondrial respiration (*n* = 4). **C** Normalization of the results showed differences in ATP coupled respiration, maximal respiratory capacity, basal mitochondrial respiration and spare capacity in FXN-deficient HAs and FXN-deficient cells + SAG when compared to the LV-scrambled-transduced cells (*n* = 4). **D** Representative energy map of all experimental conditions depicting OCR on the *y*-axis and ECAR on the *x*-axis (*n* = 4). **E** Quantification of total ATP production rate, showing the proportion of ATP that comes from glycolysis and that coming from the mitochondrial electron transport chain (*n* = 4). **F** Representative images of HAs stained with BODIPY 493/503 showing lipid droplet content and density. Scale bars: 30 μm. **G** Representative flow cytometry histogram showing lipid droplet accumulation in LV-shRNA37-transduced cells (*n* = 5). **H** Representative immunoblot and quantification of PLIN2 levels in all experimental conditions. Equal amounts of protein were immunoblotted and probed with antibodies against PLIN2 and β-actin (as loading control) (*n* = 6). Data in all graphs were normalized to the untreated group (data not plotted) and represent mean values ± S.E.M. In **E** $ refers to *p*-values of Mito ATP, whereas # refers to *p*-values of Glyco ATP. In **A**, **C**, **E**, **G**,** H**, data were evaluated by one-way ANOVA followed by Tukey’s post hoc test

Once we observed that mitochondrial morphology and dynamics were altered in HAs lacking FXN, we investigated if these changes were affecting mitochondrial activity and functioning. For this, we monitored mitochondrial superoxide production in living cells [70]. The flow cytometry analysis revealed that FXN-deficient HAs produced more mitochondrial superoxide than the LV-scrambled-transduced cells, detecting as well that SAG partially blocked this effect when administered to LV-shRNA37-transduced cells (Fig. 4A). When we examined oxygen consumption rates and bioenergetic parameters of control and FXN-deficient HAs using the Seahorse XF24, we observed metabolic changes and defects in mitochondrial respiration. We found that FXN deficiency impaired ATP coupled respiration, maximal respiratory capacity, basal mitochondrial respiration and spare capacity (Fig. 4B and C). All these parameters, except for ATP coupled respiration, were rescued after SAG treatment. Interestingly, FXN deficiency decreased both the oxygen consumption rate (OCR), which is due to mitochondrial respiration, and the extracellular acidification rate (ECAR), which is due to glycolysis, indicating that these cells have a metabolic decline when compared to control cells (Fig. 4D and E). Moreover, when FXN-deficient HAs were chronically treated with SAG, both energetic measurements returned to similar rates as those observed in control conditions, indicating a recovery of the metabolic activity.

In previous studies, intracellular lipid accumulation, a sign of altered lipolysis, has been observed in FRDA patient-derived cells [71]. In line with these data, we tested whether *FXN* silencing in astrocytes altered lipid metabolism by measuring lipid droplet accumulation in living cells. Immunofluorescence and flow cytometry studies revealed a strong accumulation of lipid droplets in FXN-deficient HAs, which is probably due to the mitochondrial dysfunction triggered by the lack of FXN in the cells. In our hands, SAG significantly reduced the accumulation of these organelles (Fig. 4F and G). To complement these results, we analyzed perilipin-2 (PLIN2), whose function involves the formation and accumulation of lipid droplets [72, 73]. We detected an increase in PLIN2 levels in HAs lacking FXN. The treatment with SAG significantly decreased PLIN2 to control levels (Fig. 4H). Altogether, these data suggest that FXN deficiency in HAs induce structural and functional defects in mitochondria. These changes involve the production of mitochondrial superoxide, variations in several respiratory parameters and the accumulation of lipid droplets, probably due to an impairment of fatty acid degradation in the mitochondria. Importantly, the majority of these changes were effectively prevented by chronically treating the cells with SAG.

### SAG modifies the astrocyte reactivity profile of FXN-deficient cells

**Fig. 5:**
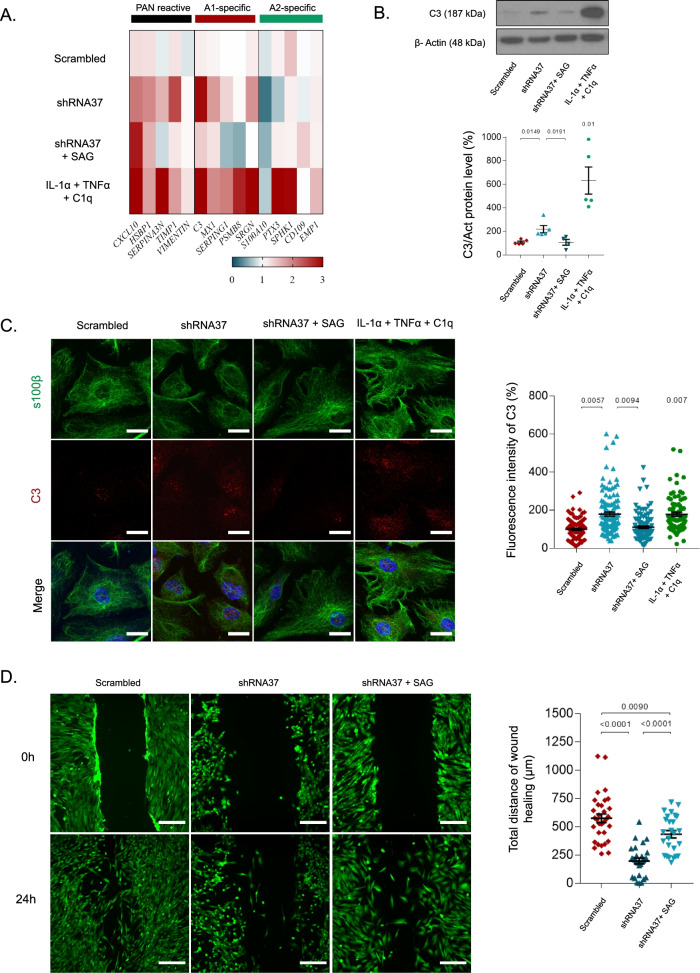
FXN deficiency induces a reactive phenotype in HAs that is attenuated by SAG. In **A–D**, HAs were cultured under standard conditions (untreated) or transduced with either the LV-scrambled or LV-shRNA37 for 96 h, SAG was added daily at 1 μM for 6 days. **A** Heat map of reactivity transcripts of all experimental groups analyzed by qPCR, split in PAN reactive, A1-specific and A2-specific reactivity genes. As positive control, HAs were treated with IL-1α, TNFα and C1q for 24 h. Gene expression was normalized to the housekeeping gene GAPDH, quantifying fold change relative to untreated group by the comparative Ct method (*n* = 4). **B** Representative immunoblot and quantification of C3 levels in all experimental groups. Equal amounts of protein were immunoblotted and probed with antibodies against C3 and β-actin (as loading control) (*n* = 5). **C** The panels on the left show representative images of HAs in the indicated experimental conditions. Cells were stained with specific antibodies against S100β and C3 proteins and with To-Pro-3 to stain the nuclei (blue). Scale bars: 20 μm. The graph on the right shows the fluorescence intensity of C3 + cells in the indicated experimental groups. Each plotted value represents a single cell from at least three independent experiments. **D** Scratch-wound assay images and quantification of HAs in the different experimental conditions from 0 to 24 h post-scratch (*n* = 4). The total migration distance was quantified from the acquired images as the distance traveled by HAs from 0 to 24 h. Scale bars: 100 μm. In all graphs, values were normalized to the untreated group (data not plotted). In **B** data represent mean values ± S.E.M while in **C, D** data represent values ± S.E.M of the acquired. In **A–D** data were evaluated using one-way ANOVA followed by Tukey’s post hoc test. In **B**, **C**, data from the IL-1α, TNF-α and C1q treated group were compared to the untreated group (data not plotted) using a one-sample *t*-test

As previously mentioned, reactive astrocytes were recently classified in at least two different phenotypes [14]. However, recent reports support the idea of moving beyond this binary vision of astrocyte reactivity, by assessing complementary molecular and functional parameters [74]. Following these recommendations, we combined different approaches to thoroughly determine the reactivity profile of FXN-deficient HAs. First, we evaluated a battery of the main markers associated with pan reactive, A1-inflammatory and A2-protective phenotypes in our model, using IL-1α, TNF-α and C1q-treated cells as a positive control for A1-reactivity. In these experimental conditions, we observed that FXN deficiency in HAs induced an upregulation of general reactivity and A1-phenotype transcripts, while transcripts of A2-phenotype were either downregulated or remained unmodified (Fig. 5A and Additional file 1: Table S2). By contrast, FXN-deficient HAs chronically treated with SAG experienced an important transcript downregulation of most general and A1-reactivity markers, while no significant changes were observed in transcripts of A2 reactivity markers. Consistent with the qPCR results, the chronic treatment with SAG reduced both protein expression levels and immunostaining of C3, which were increased after *FXN* knockdown (Fig. 5B and C).

As neurotoxic reactive astrocytes have some of their normal functions impaired [14, 75], to gain further insight on the impact that the lack of FXN has on HAs function, we analyzed astrocyte migration ability using the wound healing assay. For this, after forming a scratch-wound using a pipette tip on a confluent monolayer of HAs previously subjected to our different experimental conditions, we measured and compared the wound width at 0 h and 24 h. To rule out that wound closure was the result of differences in proliferation rather than in migration, we performed the same experiments in serum-free media, obtaining similar results (data not shown). When we estimated the difference in the wound width between these two time points, as a measure of wound closure due to cellular migration, we observed that wound closure in shRNA37-transduced cells was not as efficient as in the LV-scrambled condition. Moreover, in contrast to the shRNA37 condition, wound healing values were significantly higher in shRNA37 + SAG cells (Fig. 5D). We consider that migration differences are due to the cell status among conditions, however, we cannot rule out the possibility that differences in cell density at the beginning of the assay could affect cell migration and wound healing closure, and could have therefore influenced the results. In summary, these data demonstrate that upregulation of general and A1-reactivity markers in FXN-deficient cells is accompanied by a reduction in their wound healing ability, supporting the idea that FXN-deficient HAs acquired a reactive state and lost some of their functions. Besides, our data indicate that the activation of the SHH pathway with SAG prevented both phenomena.

### Treatment with SAG reduces the pro-inflammatory phenotype exhibited by FXN-deficient astrocytes

**Fig. 6:**
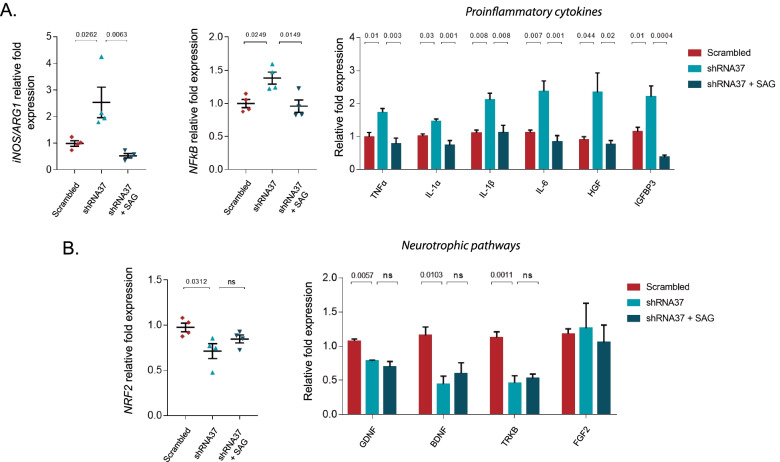
Chronic treatment with SAG diminishes the inflammatory reactivity phenotype triggered by *FXN* knockdown in HAs. In **A**, **B**, HAs were cultured under standard conditions (untreated) or transduced with either the LV-scrambled or LV-shRNA37 for 96 h, SAG was added daily at 1 μM for 6 days. **A** The graph on the left shows mRNA expression levels of *iNOS* and *ARG1* represented as the ratio of *iNOS*/*ARG1*, the graph in the middle shows expression levels of *NFkB* and the graph on the right shows expression levels of different pro-inflammatory cytokines determined by qPCR in the indicated treatments. In all cases, gene expression was normalized to the housekeeping gene GAPDH and quantified by the comparative Ct method (*n* = 4). **B** The graph on the left shows *NRF2* mRNA expression levels and the graph on the right shows mRNA expression levels of genes involved in different neurotrophic pathways, all determined by qPCR. For each gene, the expression was normalized to GAPDH and quantified by the comparative Ct method (*n* = 4). In all graphs data were normalized (data not plotted) to the untreated group and represent mean values ± S.E.M. In **A**, **B**, statistical significance was evaluated by one-way ANOVA followed by Tukey’s post hoc test

Although the precise molecular mechanisms by which astrocytes contribute to neurodegeneration are not entirely determined yet, it has been proposed that astrocytes exacerbate neuroinflammation by releasing pro-inflammatory molecules like cytokines [18, 22, 76, 77]. Nevertheless, astrocytes are important regulators of neuronal functioning, being able to protect against inflammation through multiple mechanisms, including the release of antioxidant and neurotrophic factors [78–81]. As the A1/A2 reactivity states are closely related to a pro-/anti-inflammatory status, we analyzed some signaling pathways that could be involved on the inflammatory phenotype observed in FXN-deficient HAs. To evaluate the astrocytic inflammatory response, we measured the *iNOS*/*Arginase-1* gene expression ratio, with a higher ratio of *iNOS* indicating a pro-inflammatory response, and a higher expression of *Arginase-1* indicating a shift towards an anti-inflammatory state [82]. In our experimental conditions, the *iNOS/Arginase-1* ratio was significantly higher in FXN-deficient HAs than in control conditions, and this ratio was significantly reduced after chronically treating the cells with SAG (Fig. 6A). Besides, we also observed an increased signaling via NFkB in FXN-deficient astrocytes, which diminished as well after the treatment with SAG.

In view of these results, we checked the expression of several pro-inflammatory cytokines and observed that FXN deficiency significantly upregulated *TNF-α, IL-1α, IL-1β, IL-6, HGF* and *IGFBP3* in HAs (Fig. 6A). In a similar manner to *NFkB*, the transcript levels of these cytokines were reduced after chronically treating the cells with SAG. On the other hand, when we evaluated several antioxidant and neurotrophic signaling pathways, along with a reduction in *NRF2* transcript levels, we found that FXN deficiency decreased *GDNF*, *BDNF* and *TRKB* transcripts, without inducing significant changes in *FGF2* across experimental conditions (Fig. 6B). Importantly, the chronic treatment of FXN-deficient cells with SAG was unable to revert the expression levels of any of these genes to control levels. Globally, these results suggest that FXN deficiency polarizes HAs towards a pro-inflammatory reactivity profile, and that chronic treatment with SAG reduced the inflammatory response observed in these cells.

### Neurotoxicity and synapse loss are abolished after chronically treating astrocytes with SAG

**Fig. 7:**
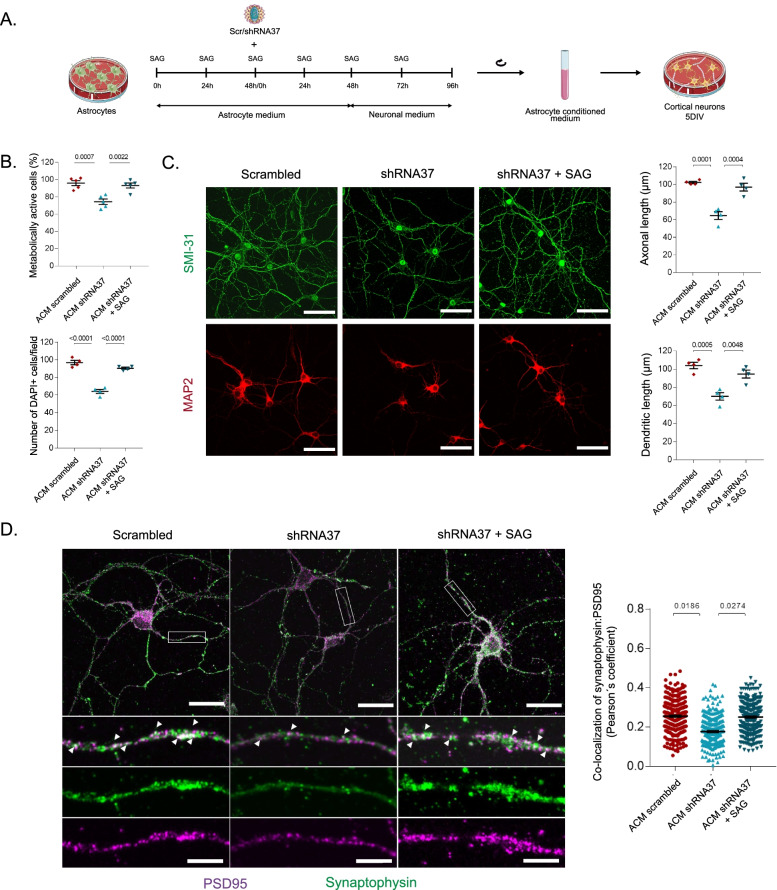
SAG attenuates neurotoxicity induced by FXN-deficient reactive astrocytes. **A** Schematic representation of ACM collection and treatment of mouse cortical neurons. For these experiments, cortical neurons were cultured for 5 days (120 h) in ACM from HAs cultured under standard conditions (untreated) or transduced with either the LV-scrambled or LV-shRNA37 and treated daily or not with SAG at 1 μM. 48 h prior to ACM collection, the medium was shifted to complete neuronal medium. **B** Quantification of the cellular metabolic activity (*n* = 3) and total cell number estimated by DAPI counts (*n* = 4) in all experimental groups of mouse cortical neurons after ACM treatment. **C** The left panels show representative confocal images of mouse cortical neurons cultured in ACM from the indicated experimental conditions, that were stained with specific antibodies against MAP2 to stain dendrites and SMI-31 to stain axons (*n* = 4). Scale bars: 50 μm. The graphs on the right show axonal and dendritic lengths calculated by measuring the total length per field corrected by the number of cells per field on each acquired image. **D** The left panels show representative confocal images of mouse cortical neurons cultured in the different ACM and stained with the presynaptic marker synaptophysin and the postsynaptic marker PSD-95 (*n* = 4). Synapse number was estimated by analyzing synaptophysin and PSD95 co-localization using the plugin JACoP (ImageJ). Insets are threefold enlargements of the boxed region. Scale bars: 25 μm (expanded fields) and 5 μm (insets). All values were normalized to the group cultured in ACM from untreated astrocytes (data not plotted). Data in all graphs represent mean values ± S.E.M. Statistical significance in **A–C** was determined using one-way ANOVA followed by Tukey’s post hoc test

In homeostatic conditions, astrocytes are essential for neuronal functioning and survival [83–86]. However, when astrocyte functions are altered, neuronal survival is compromised. To determine whether the change in the reactivity state of HAs induced by the lack of FXN has a negative impact on neurons. In these studies, neurons were cultured in conditioned media from astrocytes (ACM). Thus, we analyzed the effects of FXN deficiency in HAs over wild-type mouse cortical neurons. For that purpose, we followed the protocol described in Fig. 7A. Once that ACM was collected and used to culture cortical neurons for 5 days, we evaluated neuronal viability. We observed a significant decrease in cell viability in neurons cultured in ACM from FXN-depleted astrocytes. By contrast, ACM from FXN-deficient HAs that were treated with SAG did not result in neuronal death (Fig. 7B). To rule out the possibility that SAG was remaining in the collected ACM and this could have an effect on neurons, in parallel with the rest of the conditions, cortical neurons were treated with ACM from the shRNA37 condition, adding SAG directly to the culture. However, we did not observe any effect on the MTS assay when this molecule was directly added to neurons, indicating that the observed effects of SAG were astrocyte-mediated (Additional file 1: Fig. S1﻿). When we quantified axonal and dendritic outgrowth, we observed that neurons had a dense axonal and dendritic arborization in all conditions, except for those neurons cultured in ACM from FXN-deficient cells, where we observed significantly less neurite branching and density (Fig. 7C). We also analyzed the influence of ACM from our experimental conditions over synaptic formation, which is critically enhanced upon addition of ACM [87]. For this, neurons were stained with synaptophysin and PSD95 to visualize the presynaptic and postsynaptic contacts, analyzing the percentage of co-localization between these two markers (Fig. 7D). These analyses revealed that the number of synapses decreased in neurons cultured with ACM from FXN-deficient cells, suggesting that conditioned media from those cells impaired the correct formation of these structures. By contrast, we found that synapse formation in neurons grown with ACM from FXN-deficient astrocytes that were treated with SAG was similar to those neurons cultured in control ACM. As neurons were in contact exclusively with ACM, all the effects observed in our experiments were cell-to-cell contact independent, suggesting the release of one or more soluble factors by FXN-deficient HAs as the mechanism underlying these changes. In summary, our data suggest that FXN-deficient astrocytes become reactive and release one or more soluble factors to the media that impair neuronal viability, neurite outgrowth and synapse formation, nonetheless, all these effects can be pharmacologically blocked by activating the SHH pathway with SAG.

## Discussion

**Fig. 8:**
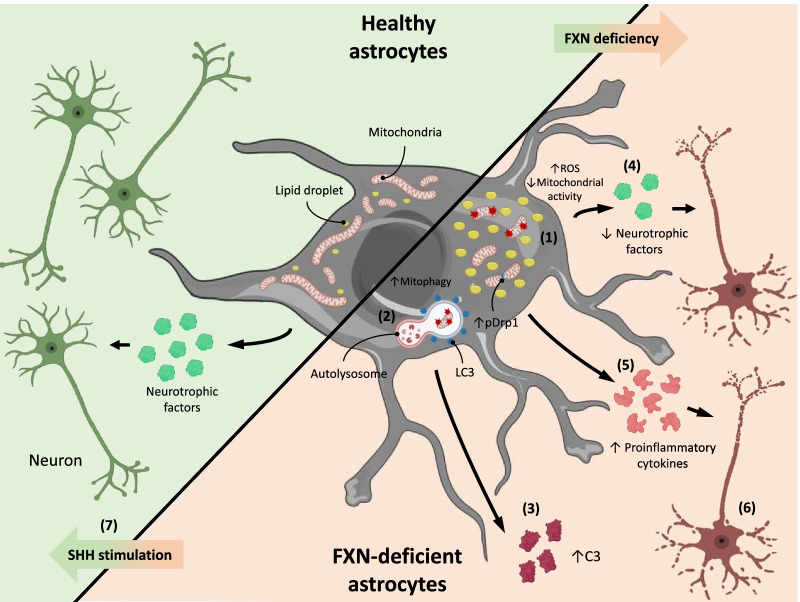
Diagram showing the effects of FXN deficiency in human astrocytes and the therapeutic effect of SAG. FXN depletion in HAs induces a series of negative effects on the cells including (**1**) changes in mitochondrial number, dynamics and function, (**2**) increased mitochondrial elimination through mitophagy, (**3**) secretion of the reactivity marker C3, (**4**) reduced production of neurotrophic factors and (**5**) higher release o of pro-inflammatory cytokines. In addition, FXN deficiency altered HA function, having a negative impact in neuronal status, i.e., reducing neuronal survival, branching and synapse formation (**6**). Most of these changes, including the indirect neurotoxic effects induced by the lack of FXN in HAs, were inhibited by a chronic treatment with SAG, a SHH pathway agonist (**7**). *FXN* frataxin, *ROS* reactive oxygen species, *C3* complement component C3, *SAG* smoothened agonist

Increasing evidence is pointing out the active contribution of glial cells, specially astrocytes, to pathological processes in several neurodegenerative diseases, including FRDA, opening up new pathways for the development of therapeutically effective strategies to treat those pathologies. Since astrocytes carry out important regulatory functions, it is highly plausible that alterations in their functions could have a significant impact in disease maintenance and/or progression [11, 12]. In this work, we characterized an in vitro model of *FXN* knockdown in HAs. This model allowed us to study the changes and alterations that pathological levels of FXN induce directly in HAs, observing as well astrocyte-mediated changes in neurons and demonstrating that the activation of the SHH pathway with SAG has beneficial effects in most of the altered parameters (Fig. 8). In specific, we found that the chronic treatment with SAG upregulated the target genes *PTCH1* and *GLI1*, indicating an activation of the pathway [24, 88, 89], and downregulated *GLI3*, which acts as an inhibitor of the SHH pathway [90]. In agreement with our last results, *FXN* knockdown in HAs induced a series of changes in the cells, i.e., less cell survival, high levels of p21, p53, cleaved PARP1 and cleaved caspase 3 [22]. Here, all the aforementioned changes where effectively abolished by chronically treating the cells with SAG. Our results are consistent with previous studies showing that activation of SHH signaling increases cellular viability [91–93].

FXN is mainly a mitochondrial protein whose function involves regulating crucial processes for maintaining a healthy mitochondrial and iron metabolism. Several studies have reported altered mitochondrial morphology and activity in different FRDA models [22, 60, 94–96]. In agreement with these reports, several of our analyses showed that upon *FXN* silencing in HAs the filamentous mitochondrial network present in healthy cells was replaced by a more fragmented mitochondrial pattern is accompanied by a significant decrease in mitochondrial mass, and signs of increased mitochondrial fission and decreased mitochondrial fusion*.* Noteworthy, similar to what occurs in hippocampal neurons, where the activation of the SHH signaling pathway elongated mitochondria [97], the treatment of FXN-deficient astrocytes with SAG restored mitochondrial mass and the fission/fusion balance. This excessive mitochondrial fission and fragmentation has been reported in other neurodegenerative diseases, confirming the importance of well-controlled mitochondrial dynamics for a correct cell function [18, 98]. Furthermore, numerous studies have demonstrated a close relationship between increased mitochondrial fission and cell death by apoptosis. In those studies, excessive mitochondrial fragmentation led to caspase activation, BAX translocation to the mitochondria and the release of cytochrome c, triggering cell death by apoptosis, a process we have shown in our astroglial FRDA model [65, 99, 100]. In our hands, the chronic treatment with SAG reduced mitochondrial fragmentation and apoptosis, and future research will determine whether these effects are due to a direct improvement in mitochondrial dynamics, or if other mechanisms are involved.

Excessive Drp1 activation induces mitochondrial fission and mitochondrial dysfunction through the dissipation of the mitochondrial membrane potential, which in turn, induces the stabilization of PINK1 in this organelle and the subsequent recruitment of Parkin, triggering mitophagy [100, 101]. We found that several of the proteins involved in autophagy and mitophagy were elevated in FXN-deficient HAs, suggesting that the observed reduction in mitochondrial mass could be due to a cytoprotective response through selectively removing damaged mitochondria [102]. It is tempting to speculate that the decrease in mitophagy observed in SAG-treated FXN-deficient HAs results from the improvement of mitochondrial function triggered by SAG. However, this hypothesis was not directly addressed in our study.

After treating FXN-deficient astrocytes with SAG, some of the mitochondrial defects that we and others have observed when FXN is depleted, like increased mitochondrial superoxide production and impaired/altered respiration, were prevented [103, 104]. Previous studies already reported an effect of SHH signaling activation on mitochondrial oxidative phosphorylation and fission [97, 105], and our results are in agreement with these observations. Our data suggest that the improvement of mitochondrial function induced by SHH activation could be preventing mitochondrial superoxide production, as previously reported in primary mouse astrocytes [106]. To the best of our knowledge, this is the first report showing that SHH agonists like SAG have beneficial effects on astrocyte mitochondrial metabolism and function.

Previously, we demonstrated that FXN-deficient HAs become reactive and release molecules that are toxic for neurons. In this study, we extended those observations and evaluated the reactivity state of astrocytes according to the recently defined subtypes of neurotoxic A1 and neuroprotective A2 [14]. As we found that all general reactivity markers and most A1-reactivity markers were upregulated in our model, while A2 reactivity markers were either downregulated or remained unchanged, our data indicate that FXN-deficient cells acquire an A1-like phenotype. Interestingly, in FXN-deficient astrocytes treated with SAG, the majority of general reactivity and A1 markers were downregulated, without having major changes in A2 markers, which means that a shift in astrocyte reactivity towards a resting state occurred. Other studies using different experimental models have found clear similarities with the canonical definition of A1 or A2 reactive astrocytes. However, these same studies have also found significant variations of these phenotypes [15, 17–20, 107]. Thus, instead of classifying astrocytes in A1 vs A2, several authors have suggested the existence of a spectrum with multiple reactivity profiles for different diseases, in which different populations of astrocytes might coexist [20]. In this context, our results are in agreement with that statement, because we have observed some similarities with the canonical A1 and A2 phenotypes, however, we have also observed important differences. For this reason, in an attempt to have an in-depth characterization and functional analysis of our model, we have also assessed some other biochemical and functional features of our FXN-deficient astrocytes.

One of the main features of neurotoxic reactive astrocytes is the upregulation of pro-inflammatory genes, and we found a pro-inflammatory state in our HAs that was accompanied by the upregulation of several pro-inflammatory cytokines. We detected that NFkB signaling was increased in FXN-deficient astrocytes. This pathway controls cytokine production and is strongly related with neuroinflammation [108, 109], and its activation has been reported in different neurodegenerative diseases [110–112] and in A1 reactive astrocytes [14, 45]. Thus, although it is not clear yet whether the activation of NFkB is necessary for astrogliosis, it seems plausible that the activation of this pathway might be involved in promoting neuroinflammation and neurodegeneration. In our model, the chronic treatment with SAG attenuated the pro-inflammatory phenotype induced by the lack of FXN in HAs by reducing NFkB levels and cytokine production. Interestingly, recent studies have found that SHH inhibits NFkB signaling and reduces apoptosis by increasing the expression of the anti-apoptotic protein Bcl-2 [93]. On the other hand, we evaluated antioxidant and neurotrophic routes that could also be affected by FXN deficiency, and studied SAG effects on them. One of the antioxidant routes that is compromised in other FRDA models is Nrf2 [113], and our data are consistent with those results. In addition, we found that *GDNF*, *BDNF* and *TRKB* were significantly altered in our model and, similar to what occurred with Nrf2, SAG was unable to increase the levels of any of these molecules. These results strongly suggest that SAG effects are more directed at reducing the inflammation present in FXN-deficient HAs rather than at activating their neurotrophic properties. However, more studies are necessary to determine the molecular mechanisms underlying SAG effects on astrocytes.

The fact that *FXN* silencing in HAs induces a loss of function in these cells, i.e., loss of migration capacity and failure to support neuronal neurite growth and synapse formation, confirms that FXN is a crucial protein for the normal function of astrocytes. Neurons cultured in ACM from FXN-deficient astrocytes showed reduced cell survival and dendritic and axonal length, hallmarks of neurodegeneration. However, when FXN-deficient astrocytes were treated with SAG, and the reactivity phenotype was attenuated, neurodegeneration was limited and synapse formation was recovered. Neuronal death has also been observed using similar experimental paradigms in other neurodegenerative disease models [12, 15, 114–116]. As throughout the experiments there was no physical contact between astrocytes and neurons, we rule out the possibility that any of the astrocyte-mediated effects we observe in neurons are cell contact-dependent. Thus, our analyses suggest that in addition to releasing pro-inflammatory mediators and reducing neuronal support, FXN-deficient astrocytes exert their neurotoxic effects by releasing one or more soluble factors. As the chronic treatment with SAG attenuates astrocyte-mediated neurodegeneration, we consider that SAG is either blocking the release of those neurotoxic factors or inducing the release of one or more soluble factors that have a positive impact on neuronal survival, as it has been recently reported in a neurodegenerative model using kainic acid [24].

One of the limitations of our study is that we used an iRNA approach to achieve similar pathological FXN levels as those found in FRDA patients. However, FXN downregulation in patients is due to the formation of aberrant DNA structures and epigenetic changes in this gene [117, 118]. Thus, while in patients this process occurs gradually, in our model we are causing an abrupt reduction in FXN levels that is probably inducing a more severe condition than the one reported in the clinical scenario. Therefore, future studies should use more physiologically relevant models to better understand the role of glial cells in the disease and the possible beneficial effect of using compounds that modulate the SHH signaling pathway.

## Conclusions

By establishing and characterizing an in vitro model of FXN depletion in HAs, here we have demonstrated that these cells are actively contributing to the neurodegenerative process associated with pathologies like FRDA. Our results highlight the multidimensional roles of astrocytes as neuroprotective or neurotoxic modulators and the idea that neuron–glia interactions are affected in the disease. We have also reported the potential that the activation of the SHH signaling pathway with small molecules like SAG, could have to revert astrocyte reactivity and neurotoxicity and to provide neuroprotection. Future research on the mechanisms by which SHH activators like SAG are regulating neuron–glia cross talk in experimental models of the disease will shed light on the possibility to modulate this pathway to target and limit/arrest neurodegeneration not only in FRDA, but also in other neurodegenerative diseases.

## Supplementary Information

**Additional file 1.** **Table S1.** Human primer sequences used for qPCR. **Table S2.** Statistical analysis of the pan, A1 and A2 reactive marker transcripts analyzed by qPCR. Values represent the mean ± S.E.M. from four biologically independent experiments. One-way ANOVA followed by Tukey's post hoc test or a 1-sample t-test were used to test for statistical significance. n.s, not significant. **Figure S1.** MTS quantification of neuronal metabolic activity upon addition of ACM. For these experiments, cortical neurons were cultured for 5 days (120 h) in ACM from HAs cultured under standard conditions (untreated) or transduced with either the LV-scrambled or LV-shRNA37, treated daily or not with SAG at 1 μM.

## Data Availability

All data used and analyzed for the current study are available from the corresponding author on reasonable request.

## Abbreviations

ACM: Astrocyte conditioned medium
FCCP: Carbonyl cyanide 4-(trifluoromethoxy) phenylhydrazone
CNS: Central nervous system
CTCF: Corrected total cell fluorescence
ECAR: Extracellular acidification rate
FXN: Frataxin
FRDA: Friedreich’s ataxia
GAPDH: Glyceraldehyde-3-phosphate dehydrogenase
HAs: Human astrocytes
MOI: Multiplicity of infection
OCR: Oxygen consumption rate
PI: Propidium iodide
shRNA: Short hairpin RNA
SAG: Smoothened agonist
SHH: Sonic hedgehog
S.E.M: Standard error of the mean
