# Supplementary material for: The smoothened agonist SAG reduces mitochondrial dysfunction and neurotoxicity of frataxin-deficient astrocytes
Source: J Neuroinflammation. 2022 Apr 12;19:93. doi: 10.1186/s12974-022-02442-w (PMC9006607; doi:10.1186/s12974-022-02442-w)
Supplement: Supplementary file 1 — Additional file 1. Table S1. Human primer sequences used for qPCR. Table S2. Statistical analysis of the pan, A1 and A2 reactive marker transcripts analyzed by qPCR. Values represent the mean ± S.E.M. from four biologically independent experiments. One-way ANOVA followed by Tukey's post hoc test or a 1-sample t-test were used to test for statistical significance. n.s, not significant. Figure S1. MTS quantification of neuronal metabolic activity upon addition of ACM. For these experiments, cortical neurons were cultured for 5 days (120 h) in ACM from HAs cultured under standard conditions (untreated) or transduced with either the LV-scrambled or LV-shRNA37, treated daily or not with SAG at 1 μM. [file 12974_2022_2442_MOESM1_ESM.docx]

**Additional file 1**

**Table S1.** Human primer sequences used for qPCR

**Table S2.** Statistical analysis of the pan, A1 and A2 reactivity markers in HAs

**Figure S1**

**
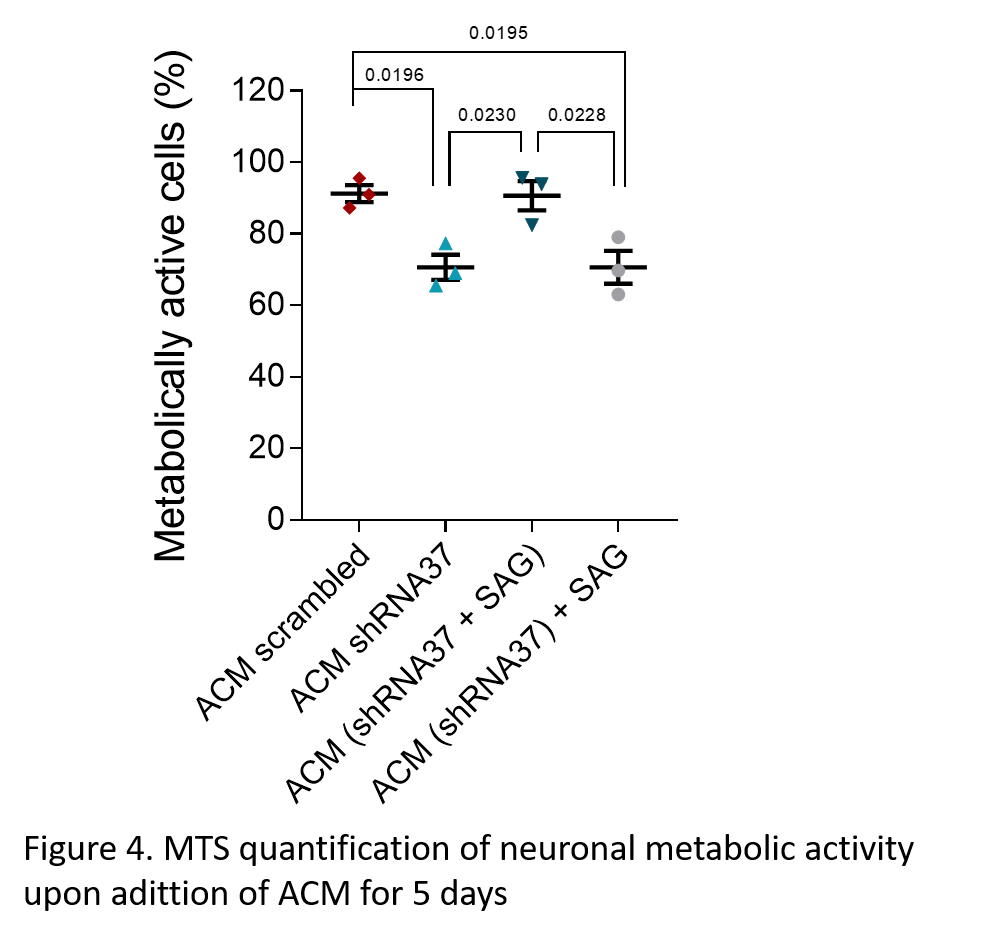
**

**Figure S1. MTS quantification of neuronal metabolic activity upon addition of ACM for 5 days**. For these experiments, cortical neurons were cultured for 5 days (120 h) in ACM from HAs cultured under standard conditions (untreated) or transduced with either the LV-scrambled or LV-shRNA37 and treated daily or not with SAG at 1 μM.
